# Case Report: Transcatheter closure of a giant asymptomatic right coronary artery-to-left ventricular fistula in a 16-year-old female

**DOI:** 10.3389/fcvm.2026.1840193

**Published:** 2026-08-14

**Authors:** Longyu Li, Yafei He, Qun Wang, Wenhua Lin

**Affiliations:** TEDA International Cardiovascular Hospital, Tianjin University, Tianjin, China

**Keywords:** adolescent, coronary artery fistula, coronary artery-to-left ventricular fistula, transcatheter closure, vascular plug, ventricular remodeling

## Abstract

A 16-year-old asymptomatic female was referred for evaluation of a single episode of self-limited sinus tachycardia (∼110 bpm) incidentally detected during a routine physical examination. Transthoracic echocardiography revealed a markedly dilated right coronary artery (15 mm) and a 7-mm fistulous tract communicating with the left ventricle. Cardiac computed tomography confirmed a right-dominant system, a tortuous, ectatic right coronary artery (maximum diameter 15 mm), and a 9-mm fistulous opening into the left ventricle. Indexed left ventricular end-diastolic diameter was significantly increased, indicating volume overload despite preserved systolic function. The patient underwent successful transcatheter closure using an 18 mm Amplatzer Vascular Plug II. At 6-month follow-up, indexed left ventricular end-diastolic diameter had normalized, and the patient remained asymptomatic. This case demonstrates that asymptomatic coronary artery fistula to the left ventricle can induce significant ventricular remodeling, which is reversible with timely intervention. Echocardiographic evidence of chamber enlargement alone should be considered an independent indication for closure.

## Introduction

Coronary artery fistulas (CAFs) are rare congenital anomalies, with those draining into the left ventricle (CAF-LV) accounting for only 3%–5% of all CAFs (1, 2). Unlike fistulas draining into the right heart, CAF-LV creates a left-to-left shunt with diastolic flow entering directly into the left ventricle, resulting in left ventricular volume overload without affecting the pulmonary circulation. This often explains the absence of audible murmurs and contributes to delayed diagnosis (2).

The management of asymptomatic CAF-LV remains controversial. While traditional teaching favored conservative observation, accumulating evidence suggests that chronic volume overload, even without symptoms, can lead to progressive left ventricular dilation, myocardial ischemia, and heart failure (3, 4). The threshold for intervention is shifting toward evidence of cardiac structural compromise identified through imaging.

We report an asymptomatic adolescent with a giant right coronary artery-to-left ventricular fistula who underwent successful transcatheter closure guided by echocardiographic evidence of left ventricular enlargement, with subsequent reversal of remodeling.

## Case report

A 16-year-old female was referred for evaluation of tachycardia incidentally detected during a routine physical examination. The tachycardia was a single, non-sustained episode of narrow-complex rhythm at approximately 110 bpm, consistent with sinus tachycardia, and did not recur. She was on no medications and reported no cardiovascular symptoms, with normal exercise tolerance. Her medical and family histories were unremarkable. Her height was 161 cm and weight 58 kg, giving a body surface area (BSA) of 1.61 m^2^.

Physical examination revealed a heart rate of 75 bpm and blood pressure of 112/68 mmHg. Cardiac auscultation demonstrated no murmurs.

**Figure 1:**
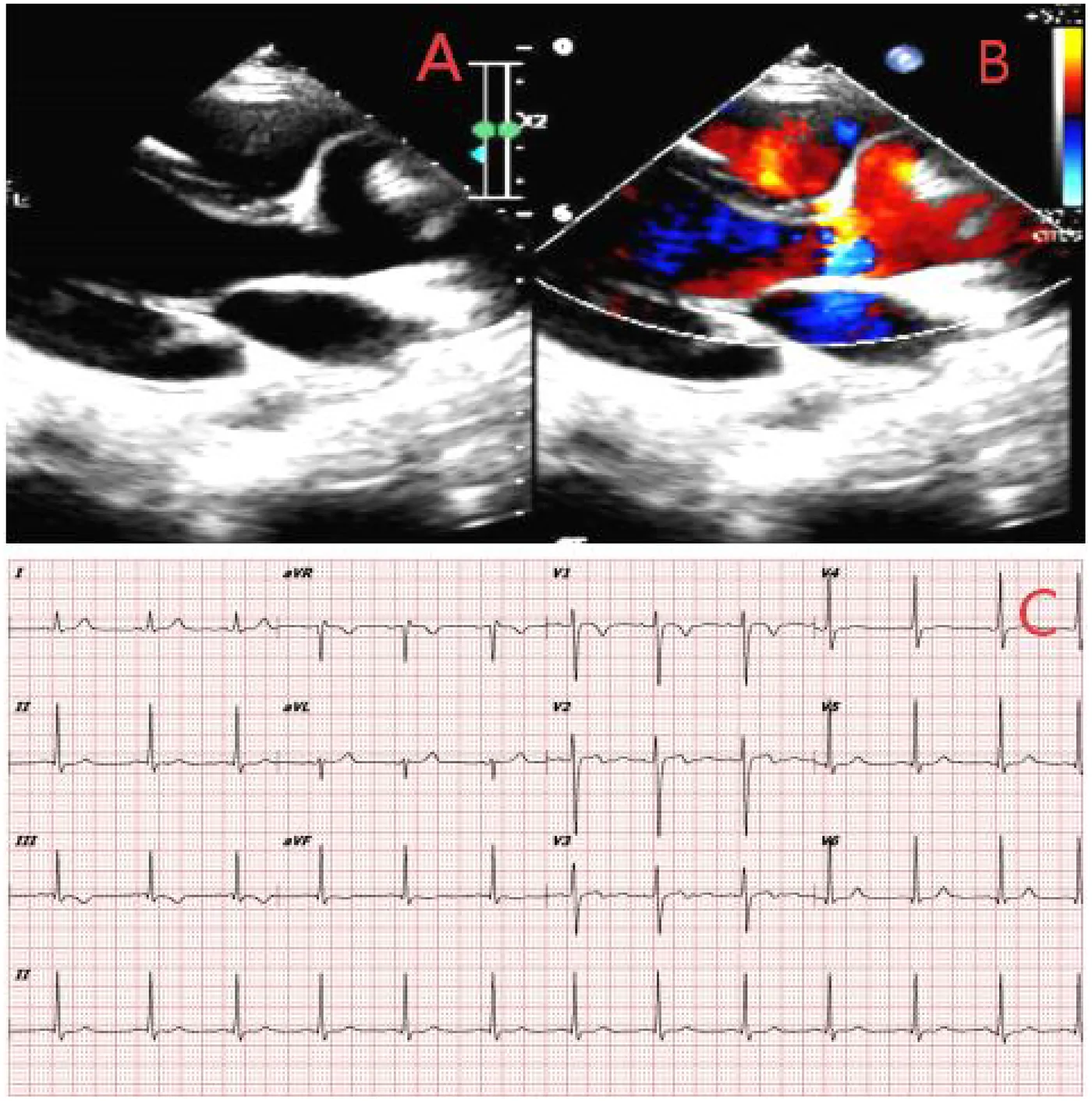
**(A, B)**echocardiography showing a markedly dilated right coronary artery. **(C)** Electrocardiography revealed non-specific ST-T changes.

Transthoracic echocardiography showed a structurally normal heart. The right coronary artery originated normally but was markedly dilated throughout its course, measuring 15 mm in diameter. A 7-mm fistulous tract originated from the distal right coronary artery and communicated with the basal inferior wall of the left ventricle. Color Doppler demonstrated high-velocity diastolic flow across the fistula (peak velocity, 3.2 m/s; peak gradient, 41 mmHg). The left ventricle was dilated (LVEDD, 57 mm; indexed LVEDD, 35.4 mm/m^2^), with preserved systolic function (LVEF, 65%). No segmental wall motion abnormalities were observed. The left coronary artery was normal (Figures 1A,B).

**Figure 2:**
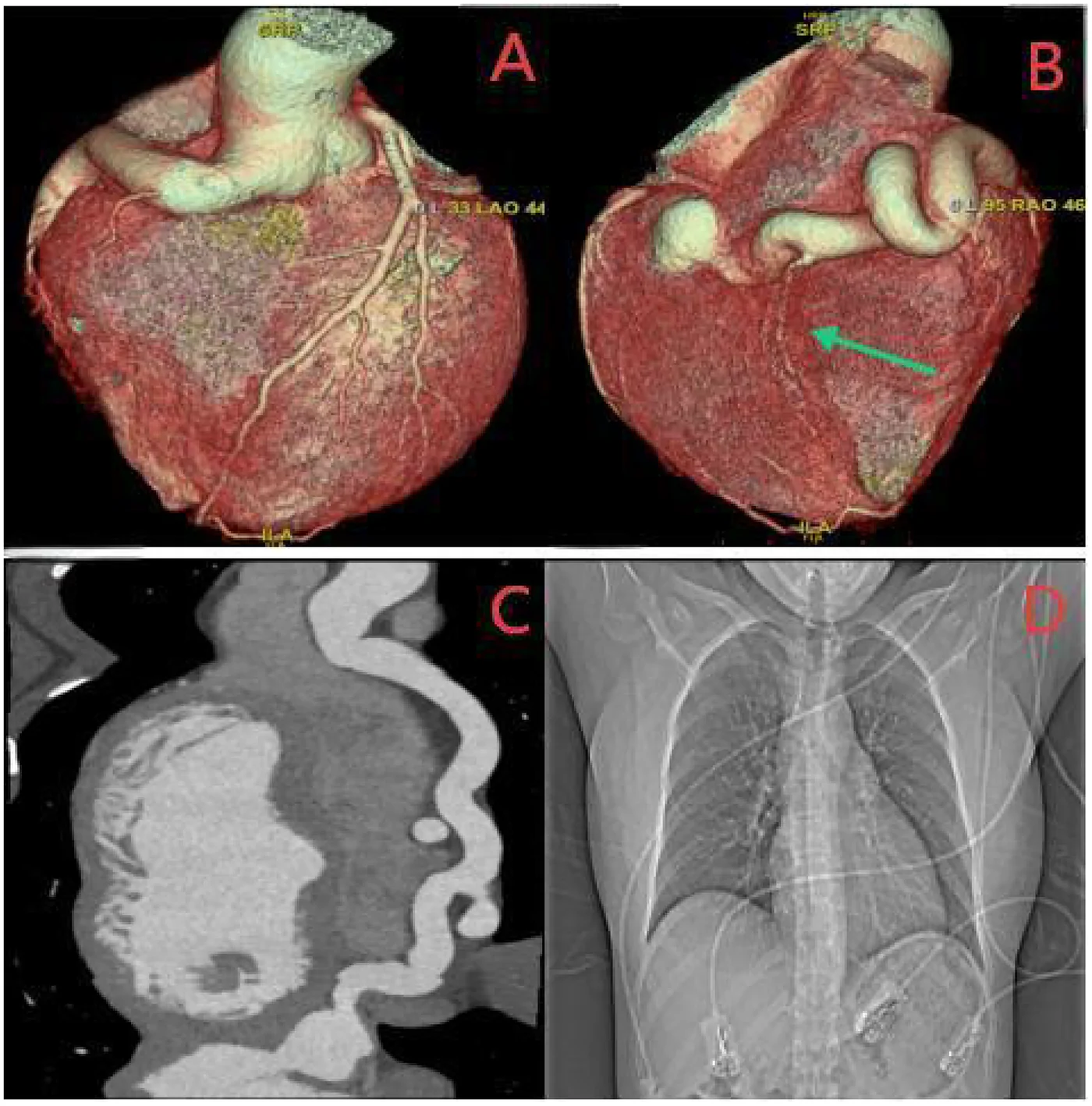
**(A—C)** Right Coronary artery-to-left ventricular Fistula. Green arrow: PDA in relation to the coronary artery fistula. **(D)** Chest radiograph was unremarkable.

Cardiac computed tomography (CT) confirmed a right-dominant coronary system. The right coronary artery was tortuous and ectatic, reaching a maximum diameter of 15 mm, and its terminal portion drained into the left ventricular cavity through a fistulous orifice measuring approximately 9 mm at the posterior left ventricular wall. **The posterior descending artery (PDA) was noted to originate in close proximity to this fistulous orifice.** All other coronary arteries were patent without significant stenosis. The left ventricle was enlarged but had normal wall thickness and homogeneous myocardial density (Figures 2A–C).

Electrocardiography on admission showed sinus rhythm with T-wave inversion in lead III and flat T waves in lead aVF, without ST-segment deviation (Figure 1C). Consistent with sinus tachycardia by history, these findings were stable and showed no dynamic changes on post-procedural day 1 and at 1-month follow-up. Chest radiography was normal (Figure 2D). Laboratory investigations, including NT-proBNP and troponin I, were within normal limits.

Given the clear evidence of left ventricular volume overload with chamber dilation on echocardiography, a multidisciplinary team concluded that intervention was indicated despite the absence of symptoms. Additional stress testing or 24-hour Holter monitoring was not performed, as the decision was driven by unequivocal structural findings and the tachycardia was an isolated, non-recurrent event with no clinical suspicion of ongoing arrhythmia.

**Figure 3:**
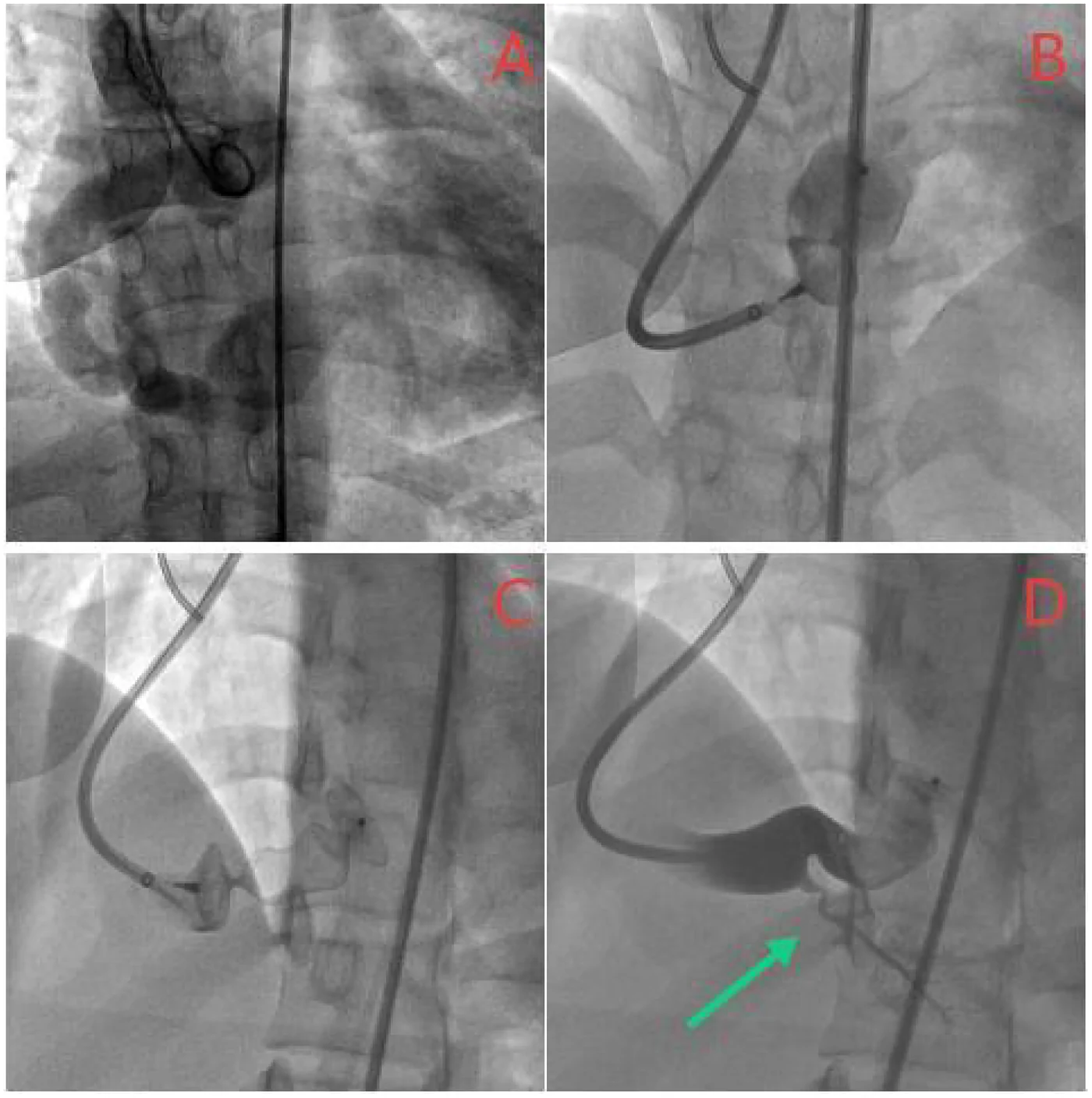
(A–D) Interventional Procedure. **(A)** Coronary angiography. **(B)** Device deployment. **(C)** Balloon occlusion test. **(D)** Angiography after device release. The green arrow points to the posterior descending artery (PDA).

Transcatheter closure was performed via right femoral arterial access under local anesthesia with conscious sedation. Cardiac catheterization confirmed a massively dilated right coronary artery with a fistulous connection to the left ventricle via a short, wide tract at the basal inferior wall (Figure 3A). A 6-French guide catheter was advanced into the right coronary artery, and a 0.035-inch guidewire was navigated across the fistula into the left ventricle. Coronary angiography and coronary CT showed no major coronary branches distal to the intended landing zone. Balloon occlusion test was performed, with no ST-T changes on ECG and no chest pain experienced by the patient. After advancing a 12-French delivery sheath, an 18 mm Amplatzer Vascular Plug II was deployed (Figures 3B,C). The device was intentionally oversized (2.0 times the 9-mm CT-measured orifice) to ensure secure anchoring in the high-flow, short-landing-zone fistula. Post-deployment angiography demonstrated a stable device position with only a trace residual shunt (Figure 3D).

Post-procedurally, high-sensitivity troponin I remained normal (<0.04 ng/mL) at 6 and 24 h, and echocardiography confirmed the absence of new segmental wall motion abnormalities, effectively excluding periprocedural myocardial injury. Given the markedly dilated right coronary artery with anticipated slow flow post-closure and the thrombogenic potential of the large device, dual antithrombotic therapy was initiated with warfarin (target INR 2.0–2.5) and clopidogrel 75 mg daily for the first 3 months, followed by aspirin 100 mg daily long-term.

Echocardiography on post-procedural day 1 showed an immediate reduction in LVEDD from 57 mm to 54 mm. At 2-month follow-up, LVEDD had decreased to 52 mm (LVEF, 63%). At 6-month follow-up, LVEDD was 51 mm (indexed, 31.7 mm/m^2^; LVEF, 65%). The right coronary artery diameter decreased only mildly, from 15 mm to 14 mm. Device position remained stable with a trace residual shunt. Mild aortic regurgitation was noted but was hemodynamically insignificant and is being monitored. The patient remained asymptomatic throughout the follow-up period (Table 1). CT angiography is planned at 12 months.

**Table 1:** Echocardiographic follow-up data.

Parameter	Pre-procedure	Post-day 1	2 Months	6 Months
LVEDD (mm)	57	54	52	51
LVEF (%)	65	64	63	65
Right coronary artery ostium diameter (mm)	15	15	14	14
Aortic regurgitation	None	None	None	Mild

## Discussion

This case addresses the controversial question of whether asymptomatic patients with CAF-LV should undergo intervention. The traditional conservative approach is increasingly challenged, particularly for CAF-LV where continuous diastolic shunting into the high-pressure left ventricle leads to insidious, progressive dilation (2, 3). The 2018 AHA/ACC guideline supports intervention not only for symptoms but also for evidence of significant hemodynamic compromise, including ventricular volume overload and chamber enlargement (4). Our patient met these criteria despite being asymptomatic, and the normalization of indexed LVEDD from 35.4 to 31.7 mm/m^2^ at 6 months confirms that such remodeling is reversible if corrected in a timely manner, before the onset of irreversible myocardial fibrosis.

The hemodynamics of CAF-LV differ fundamentally from fistulas draining into the right heart. By directly increasing left ventricular preload without affecting pulmonary circulation, CAF-LV often presents without audible murmurs, leading to delayed diagnosis (2). Chronic volume overload can progress to systolic dysfunction and heart failure (2, 3). Furthermore, the coronary steal phenomenon may cause myocardial ischemia even without epicardial stenosis (5). The stable, non-specific ST-T changes observed on our patient's ECG may reflect such subclinical steal; their lack of evolution post-procedure and normal troponin argue against any acute procedural ischemic event.

Careful device selection was critical given the fistula's dimensions (9 mm on CT) and the 15-mm aneurysmal coronary artery. The Amplatzer Vascular Plug II is well suited to this anatomy, offering high occlusion rates, precise positioning with recapturability, suitability for short and wide fistulous tracts, and rapid endothelialization (6). The decision to use an 18 mm device—2.0 times the fistula orifice diameter—was deliberate, providing the radial support necessary for stable anchoring in a high-flow lesion and mitigating embolization risk. Pre-deployment angiography clearly delineated the relationship of the landing zone to the posterior descending artery, confirming the absence of major distal branches **within the intended landing zone** and supporting the safety of the chosen position, which was consistent with the uneventful balloon occlusion test. This anatomy also meant a low risk of device-related segmental myocardial infarction, a concern further excluded by post-procedural troponin and wall motion findings.

Although the acute procedural assessment showed no immediate compromise of the posterior descending artery (PDA), we recognize that long-term device-related effects—including endothelialisation, vascular remodelling, or thrombus extension—could potentially affect this branch, especially given its proximity to the proximal disc of the Amplatzer Vascular Plug II. To mitigate this risk, we adopted a stringent dual antithrombotic regimen (warfarin plus clopidogrel for 3 months, followed by lifelong aspirin) and scheduled a 12-month coronary CT angiography to precisely evaluate PDA patency and device position. This follow-up protocol will provide critical evidence on the long-term safety of transcatheter closure in such anatomies and guide future management if any compromise is detected.

We also compared transcatheter versus surgical closure for CAF-LV. Surgery offers definitive closure but has higher morbidity, longer recovery, and a sternal scar. Transcatheter closure with the Amplatzer Vascular Plug II is less invasive, avoids cardiopulmonary bypass, and is ideal for short, wide fistulous tracts without distal branches, as in our case. Limitations include trace residual shunt, need for a 12-Fr sheath, and embolization risk. Given the patient's age and anatomy, a multidisciplinary team chose the percutaneous approach.

Post-procedural antithrombotic management for such cases lacks established guidelines. Our dual-therapy strategy—warfarin plus clopidogrel for 3 months, followed by lifelong aspirin—was tailored to the high thrombotic risk created by stagnant flow in the ectatic coronary artery and the large device surface. The absence of thrombotic events at 6 months supports the safety of this approach, though the optimal regimen remains uncertain. The mild aortic regurgitation noted at follow-up may be related to altered flow dynamics or procedural factors and is being managed conservatively (7).

A noteworthy finding is the divergent remodeling response: left ventricular reverse remodeling was robust and prompt, while the right coronary artery diameter decreased only minimally (from 15 to 14 mm). This differential response may reflect measurement limitations of echocardiography in a tortuous, ectatic vessel, the need for longer follow-up to observe vascular contraction, or, more concerningly, the possibility of irreversible structural changes in the vessel wall due to chronic hemodynamic stress (8). Definitive assessment by follow-up CT angiography is planned.

The existing literature, though limited to case reports and small series, contextualizes our findings. Successful transcatheter closure of CAF-LV using the Amplatzer Vascular Plug II has been reported in children and adolescents (9, 10), as well as in adults with giant fistulas (11). However, detailed quantitative data on left ventricular reverse remodeling are largely absent, and the long-term behavior of the dilated native coronary artery after shunt elimination remains poorly characterized. Our case contributes to filling these gaps by providing complete 6-month remodeling metrics and by documenting the discordance between rapid ventricular normalization and sluggish coronary involution. Additionally, the heterogeneity of post-procedural antithrombotic strategies across reports—ranging from short-term single-agent regimens to our dual-therapy bridge—reflects the lack of consensus and highlights the need for risk-stratified protocols.

We acknowledge the limitations inherent to a single case report. Pre-procedural Holter and stress testing were not performed, as intervention was indicated based on structural criteria alone and the tachycardia was an isolated finding. Longer follow-up is essential to confirm sustained remodeling, monitor aortic regurgitation, and definitively assess coronary artery changes (12, 13).

## Conclusion

This case demonstrates that significant, yet reversible, left ventricular enlargement can occur in asymptomatic CAF-LV. Timely transcatheter closure using an appropriately oversized Amplatzer Vascular Plug II is safe and effective. Echocardiographic evidence of ventricular dilation alone should be considered a strong, independent indication for intervention to prevent irreversible myocardial damage. While left ventricular reverse remodeling is robust, changes in the native coronary artery may be more limited, warranting ongoing surveillance—particularly for the posterior descending artery, whose long-term patency will be closely monitored by scheduled coronary CTA.

## Data Availability

The raw data supporting the conclusions of this article will be made available by the authors, without undue reservation.
